# NIRS-Based Hyperscanning Reveals Inter-brain Neural Synchronization during Cooperative Jenga Game with Face-to-Face Communication

**DOI:** 10.3389/fnhum.2016.00082

**Published:** 2016-03-08

**Authors:** Ning Liu, Charis Mok, Emily E. Witt, Anjali H. Pradhan, Jingyuan E. Chen, Allan L. Reiss

**Affiliations:** ^1^Center for Interdisciplinary Brain Sciences Research, Department of Psychiatry and Behavioral Sciences, School of Medicine, Stanford UniversityStanford, CA, USA; ^2^Program in Human Biology, Stanford UniversityStanford, CA, USA; ^3^Department of Molecular and Cell Biology, University of CaliforniaBerkeley, CA, USA; ^4^Department of Radiology, Stanford UniversityStanford, CA, USA; ^5^Department of Electrical Engineering, Stanford UniversityStanford, CA, USA

**Keywords:** functional near-infrared spectroscopy, fNIRS, hyperscanning, cooperation, obstructive interaction, inter-brain neural synchronization (INS)

## Abstract

Functional near-infrared spectroscopy (fNIRS) is an increasingly popular technology for studying social cognition. In particular, fNIRS permits simultaneous measurement of hemodynamic activity in two or more individuals interacting in a naturalistic setting. Here, we used fNIRS hyperscanning to study social cognition and communication in human dyads engaged in cooperative and obstructive interaction while they played the game of Jenga™. Novel methods were developed to identify synchronized channels for each dyad and a structural node-based spatial registration approach was utilized for inter-dyad analyses. Strong inter-brain neural synchrony (INS) was observed in the posterior region of the right middle and superior frontal gyrus, in particular Brodmann area 8 (BA8), during cooperative and obstructive interaction. This synchrony was not observed during the parallel game play condition and the dialog section, suggesting that BA8 was involved in goal-oriented social interaction such as complex interactive movements and social decision-making. INS was also observed in the dorsomedial prefrontal cortex (dmPFC), in particular Brodmann 9, during cooperative interaction only. These additional findings suggest that BA9 may be particularly engaged when theory-of-mind (ToM) is required for cooperative social interaction. The new methods described here have the potential to significantly extend fNIRS applications to social cognitive research.

## Introduction

A pervasive challenge in social neuroscience is how to model everyday human interactions in the laboratory (Hari and Kujala, 2009). The technique of hyperscanning has enormous potential to address this problem by enabling simultaneous recording of brain function in multiple interacting subjects (Babiloni and Astolfi, 2014). Hyperscanning paradigms provide a valuable platform for observing neural signatures of social cognition during social interaction. The first hyperscanning study can be traced back to an electroencephalography (EEG) study in 1965 (Duane and Behrendt, 1965). More recently, researchers have applied this technique with a variety of neuroimaging modalities, including functional magnetic resonance imaging (fMRI; Montague et al., 2002), functional near-infrared spectroscopy (fNIRS; Funane et al., 2011; Cui et al., 2012), magnetoencephalography (MEG; Baess et al., 2012) and EEG (Babiloni et al., 2011; Kawasaki et al., 2013). NIRS-based hyperscanning is a technique in which single or multiple instruments are used for simultaneous measurement of brain activity in two or more people (Scholkmann et al., 2013; Balconi and Molteni, 2015). Compared with other functional imaging techniques, fNIRS hyperscanning has the advantage of being cost-effective, portable, and more tolerant to movement. Furthermore, fNIRS allows measurement of brain activity in environments with greater ecological validity, an especially important advantage, as studies have shown that simulated tasks do not always generate the same brain activity as they do in their real-life settings (Okamoto et al., 2004). As such NIRS is increasingly considered an emerging tool in social cognitive neuroscience that can be used in a natural context with free hand movement and face-to-face oral communication. In particular, this technology offers an easy to apply and reliable brain imaging technology for measuring inter-personal interactions.

NIRS-based hyperscanning has been employed by a number of researchers to investigate interactive social behavior. For instance, Cui et al. (2012) studied the interpersonal coherence in the superior frontal cortex between two players while they played a computer-based cooperation game side by side; Holper et al. (2012) investigated the between-brain connectivity of premotor cortices in paired subjects during imitation performance of a paced finger-tapping task; the same group of people also showed a significant increase in between-brain coherence during joint n-back task performance as compared to a baseline condition (Dommer et al., 2012); Jiang et al. (2012) studied the left frontal cortices of paired participants during face-to-face communication, and showed a significant increase in the neural synchronization in the left inferior frontal cortex during face-to-face dialog between partners; the same group also investigated interpersonal neural synchronization in the left temporo-parietal junction during a group discussion among triads, and showed that the inter-brain neural synchrony (INS) for the leader-follower pairs was higher than that for the follower-follower pairs (Jiang et al., 2015); Liu et al. (2015c) studied the right inferior frontal gyrus of paired participants as they played a turn-taking (game builder or partner) game. The builder in the cooperation condition showed higher activation than his/her partner, though the same builder in the competition condition showed lower activation than in the cooperation condition. In many of these studies, interpersonal or INS has been used to denote the Wavelet Transform Coherence (WTC) of measured fNIRS signals between paired participants, in most cases, using oxy-hemoglobin concentration changes.

An important issue in fNIRS hyperscanning concerns how to spatially register cortical location data in dyadic and group analyses. A conventional way to determine the spatial location of an fNIRS measuring channel is to follow the 10–20 system, which requires an accurate placement of optodes across subjects. To date, fNIRS hyperscanning studies have solely used channel-wise analysis, which assumes that the location of each channel across subjects is representative of a common anatomical location. This approach assumes that using identical channels for both inter-brain and group analyses is methodologically valid. However, this is often not the case when placing fNIRS probes on the heads of individual subjects. Although, no single method has been broadly accepted to address this issue in hyperscanning studies, several approaches have been described for fNIRS data spatial registration of group data (Tsuzuki and Dan, 2014). For example, one suggestion is to implement image reconstruction algorithms based on the solution of the inverse problem for light transport, and to map fNIRS data to a three-dimensional voxel space, allowing voxel-wise analytic approaches (Tsuzuki et al., 2012). However, this method is primarily intended for use with a high-density fNIRS system and requires an MRI image for each subject. Another approach is to identify a ROI based on an anatomical atlas (Okamoto et al., 2009; Yanagisawa et al., 2010). In this approach, center coordinates are designated and neighboring channels or voxels are extracted to represent the functional status of a ROI. However, as Tsuzuki and Dan point out (Tsuzuki and Dan, 2014), setting a ROI by integrating channels or voxels is equivalent to spatial smoothing, and thus different ROI sizes result in different degrees of spatial filtering.

The study presented here was designed to investigate neural synchrony using fNIRS in a naturalistic, interactive setting during which subjects are able to orally communicate face-to-face while playing a non-computerized game, Hasbro’s Jenga™. Jenga has been used in various studies in social psychology, such as friendship (Wright et al., 2002; Page-Gould et al., 2008), the study of social-interactive behavior of children with social deficits (Kawaguchi et al., 2010), and psychoeducation (Briggs et al., 2011). In our study, we created conditions for the interacting dyads that could be implemented in a naturalistic manner. Three different levels of cooperation were used: full cooperation, parallel game play, and obstructive interaction. As consistent with previous descriptions (OPTIC, Paulson, 1974), full cooperation incorporates the highest level of interpersonal cooperation, while obstructive interaction is considered to be least conducive for cooperation. Previous hyperscanning studies of turn-based interdependent behaviors (Liu and Pelowski, 2014) have shown inter-brain synchronization during both cooperative and competitive interactions (Jiang et al., 2015; Liu et al., 2015b), but not in independent play (Cui et al., 2012). Thus, we hypothesized that we would observe inter-brain neural synchrony (INS) during both cooperation and obstructive interaction conditions, but not during parallel play condition. For the imaging data analysis, instead of using channel-wise analysis, we developed a new method to identify synchronized channels within a dyad and a structural node-based spatial registration approach for inter-dyad analyses.

## Materials and Methods

### Participants

Eighteen healthy volunteers (age 21.1 ± 1.7 years) participated in the study. Standardized phone interviews were conducted to screen for handedness (Edinburgh Handedness Inventory) and medical/psychiatric history. None of the subjects that participated in the study reported a history of significant medical, neurologic or psychiatric illness. All subjects were right-handed college students, and were already familiar with the game of Jenga™. Unacquainted subjects were formed into nine pairs (female/female, *n* = 2; male/male, *n* = 2; and male/female, *n* = 5). The study protocol was approved by the Stanford University Institutional Review Board. Written informed consent was obtained from all subjects.

### Task

The task was comprised of three experimental conditions: cooperation, parallel play, obstructive interaction and the control section: dialog. The dialog section was designed to control for neural activation resulting from oral communication. The order of the three task conditions was counterbalanced across dyads. During the cooperation, parallel play, and obstructive interaction conditions, participant dyads played the game of Jenga according to different objectives.

In a conventional Jenga game, players remove wooden blocks from a stacked tower formation and place it on the top of the tower. Moving the wooden blocks from the lower portion of the tower to the top will make the tower less stable. The conventional goal of the game is to keep the tower standing without falling. In our study, we introduced additional rules to structure the cooperation and obstructive interaction during the game. The general game rule for the cooperation and obstructive interaction conditions was that players took turns removing a single wooden block from a single stacked tower followed by placing the block on the top of the tower. Each player was allowed to touch and move only one block during his or her turn.

The specific rules for each experimental conditions and the dialog section are further described as follows:

(1) During the cooperation condition, dyads were instructed to work together to build the tallest possible tower in the time allotted. They took turns making a move, and were instructed to talk to one another about strategy and agree on which block to move before they made any physical movement of the wooden block. For instance, player 1 would be the player in-turn in a trial. Player 1 might propose moving a middle block on the third floor, while player 2 would advise that moving the middle block on the fifth floor would be better. The two players would then need to discuss and agree on a specific block and movement before player 1 could make any movement.
(2) During the obstructive interaction condition, dyads were instructed to adopt a strategy with their partner in order to increase the likelihood that the tower would fall. As in the cooperation condition, subjects took turns making a move, and were asked to talk about each move; however they did not have to provide truthful advice, nor listen to their partner’s recommendations. For instance, player 2 would be the player in-turn in a trial. Player 2 might propose moving a middle block on the fifth floor, while player 1 would suggest moving the left block on the third floor. Player 2 would surmise that moving the block on the third floor would make the tower fall, so player 2 would move the middle block on the fifth floor anyway without following the suggestion of player 1.
(3) During the parallel play condition, subjects played Jenga in the same area, built separate towers, with the goal of building a tower without falling. During this condition they narrated their moves to themselves, explaining their strategy, but did not interact with each other. Thus, the subject’s attention was focused primarily on his/her own materials.
(4) During the dialog section, dyads did not play Jenga, but mimicked task conversation by discussing a given topic and conversing in a back and forth manner with short sentences.

**Figure 1:**
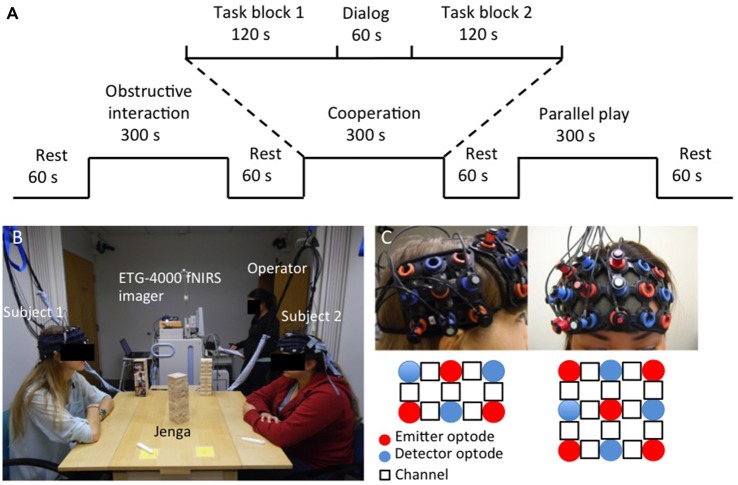
**Task design (A), experimental setup (B) and probe configuration (C)**.

Each task condition was divided into two 120 s blocks that were separated by a 60 s dialog block. A 60 s rest period was used when switching between task conditions. There were also 60 s rest periods before the first task condition and after the last task condition that served as a baseline state. Figure 1A shows an example of the task design as follows: Rest, Obstructive block, Dialog, Obstructive block, Rest, Cooperation block, Dialog, Cooperation block, Rest, Parallel Play block, Dialog, Parallel Play block, Rest. The given dialog topic between the two cooperation blocks was to “Talk about your favorite foods.” In between the two obstructive interaction blocks, the prompt was to “Talk about the courses you’ve taken.” In between the two parallel play blocks, the prompt was to “Talk to yourself about your day.”

### Experimental Procedure

The experiment was conducted with the dyad and two research staff members in one room. One staff member operated the NIRS machine while the other introduced the task and experimental procedure. The two subjects comprising a dyad were seated comfortably on opposite sides of a table facing each other, with their forearms resting on the table to minimize excess arm and neck movement. During the cooperative and obstructive interaction conditions, the Jenga tower was placed in the center of the table so that each subject could see two faces of the tower and had a clear view of his or her partner’s moves (Figure 1B). During the parallel play condition, a tower was placed in front of each subject. The tower was blocked from subjects’ view during all rest periods and dialog blocks to prevent them from working on the tower during those periods. Before the task started, subjects listened to the examiner’s verbal task instructions and watched video demonstrations of each condition. Additionally, subjects had a chance to practice the task to ensure they understood the instructions. Subjects were designated as “1” and “2” prior to the start of the experiment and subject 1 was instructed to make the first move every time a new condition began. Once the experiment started, subjects were not permitted to talk to the research staff members. After the task was complete, subjects completed a questionnaire to describe the strategies that used during the cooperative and obstructive interaction conditions to ensure they were following instructions.

### Videotaping and Data Coding

For exploratory purposes, the performance of five dyads was fully videotaped for later data coding. A camcorder was place on a side table at 3 feet 6 inches above the floor, and 3 feet away from the game table. The recorder was turned on when the task started, and turned off when the entire experiment was complete.

After videotaping was completed, we determined the onset time for each subject to move the wooden blocks. Since each subject took turns to move the blocks, the social interaction frequency was determined as the inverse of the time interval between two continuous movements of the block.

### fNIRS Data Acquisition

fNIRS signals were acquired in the two subjects simultaneously using an ETG-4000 Optical Topography system (Hitachi Medico Co., Tokyo, Japan) with a sampling rate of 10 Hz. The measurement patches consisted of an evenly distributed array of alternating emitter and detector fiber bundles (optodes). Optodes were spaced 30 mm apart, resulting in a spatial resolution of 30 mm for the system. A channel represented the area measured by one emitter-detector pair, and the channel location was defined as the center position of the emitter-detector optodes. A single “3 × 3” measurement patch containing nine optodes was positioned over the right prefrontal cortex (rPFC) of each subject’s head, resulting in 12 measurement channels. A single “3 × 2” measurement patch consisting of six optodes was positioned over the right superior temporal sulcus region (rSTS) of each subject’s head, resulting in seven measurement channels (Figure 1C). We chose these two regions based on their previously identified roles in social cognitive processes. Specifically, our previous study showed that increased coherence occurred in the rPFC of dyads during a cooperative computer task (Cui et al., 2012). Other game-based EEG studies also reported increased interbrain synchronization in the prefrontal cortex during cooperative decision-making (Astolfi et al., 2009). The rSTS has been implicated as a critical structure for processing socially relevant information, such as body or facial movement (Allison et al., 2000).

The patch placement was based on the 10–20 system. Specifically, the inside edge of the “3 × 3” patch was aligned to the midline (i.e., the arc running from the nasion through Cz to the inion), and the bottom row of the patch was right on top of the subjects’ eyebrows. The inside edge of the “3 × 2” patch was placed above the right ear (T_3_), and the bottom row was parallel to the floor and in line with T_3_–T_5_. Within participant pairs, patches were also examined and adjusted to ensure similarity of position based on the best judgment of the operator. The locations of the optodes on the head were obtained by a 3D magnetic digitizer system (PATRIOT, Polhemus, Colchester, VT, USA) right after the task was completed, which measured the placement of each optode in relation to five reference points on the subject’s head (nasion, left and right preauricular points, vertex, and inion).

### Single Subject fNIRS Data Pre-Processing

The recorded changes in optical density were first converted into concentration changes of oxy-hemoglobin and deoxy-hemoglobin using the modified Beer-Lambert law (Delpy et al., 1988). An fNIRS channel was determined to be “noisy” if the heartbeat was not identifiable in the corresponding wavelet transform map (Liu et al., 2015a), and all noisy channels were excluded from subsequent analyses. The channel-wise individual fNIRS data was registered to a standard MRI brain template (MNI152). Specifically, the channel positions in Cartesian space were first converted to the Montreal Neurological Institute (MNI) standardized space (Singh et al., 2005), and then projected to the cortical surface (Cui et al., 2011). The obtained MNI coordinates at the cortical surface can either be plotted in a 3-D space (using the plot3 command in MATLAB), or on a rendered brain template (using xjView toolbox^1^). For representation purposes, after projecting the channel position to the cortical surface, we drew a spherical region around the projection point with a radius of five voxels (~17.5 mm), and removed any portion of the sphere that fell outside the brain mask, only keeping the voxels inside the brain (Cui et al., 2011). This way, we could signify the spatial resolution of the system (3 cm).

### Paired Subjects fNIRS Data Analysis

Although attempts to place the measurement patches in a consistent manner were always made, the corresponding topography of optodes across subjects comprising a dyad did not always correspond to the same specific location. Thus, corresponding channels did not always represent the exact same brain region across a dyad. This mismatch is a common issue in fNIRS studies. Accordingly, depending on paired (numbered) channels in dyads for performing data analyses would not necessarily establish optimal correspondence between like brain regions.

**Figure 2:**
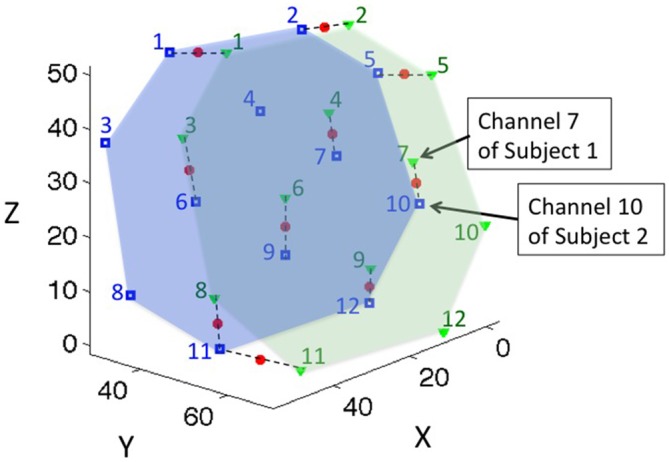
**The location of coherence for a representative dyad.** Green triangles represent the channel locations of subject 1; the green numbers label the channel indices; and the green area represents the probe coverage of subject 1. Blue squares and numbers represent the channel locations and indices of subject 2, and the blue area represents the probe coverage of subject 2. Channels were paired to their closest neighboring points within 15 mm distance, and the middle points of the paired channels were defined as the location of the coherence, represented by red dots. Dashed lines connect the paired channels for representative purposes.

To address this issue, we sought to determine the channel pairs that represented the same brain location across members of each individual dyad. Considering the spatial resolution of the fNIRS system, we defined corresponding channels as those that were within 15 mm of the same brain location. We first projected the channel positions of both subjects on a common 3-D space (Figure 2). We then paired the channels of subject 1 to their closest neighboring channels of subject 2. If the closest neighboring channel was more than 15 mm away, they were not paired. We called channels pairs within the 15 mm criterion “effective channel pairs”. We also determined the MNI coordinates of the middle point of each effective channel pair by using linear interpolation, and referred to that point as the “coherence channel” for the dyad.

Inter-subject coherence analysis was performed for each effective channel pair using the WTC package developed by Grinsted et al. (2004), Chang and Glover (2010) and custom MATLAB code (MathWorks). WTC is a method of measuring the cross-correlation between two time series as a function of frequency and time (Torrence and Compo, 1998), and was first applied to fNIRS hyperscanning in an earlier study from our group (Cui et al., 2012). Since then, other research studies have successfully applied WTC methodology to fNIRS data analyses (Dommer et al., 2012; Holper et al., 2012; Jiang et al., 2012, 2015). Thus, using WTC to characterize inter-brain synchronization, two oxy-Hb time series were obtained from each effective channel pair of each dyad. A frequency band was identified to further analyze the task effect. The coherence value in this band was averaged and the resulted single time series was assigned to the corresponding coherence channel.

**Figure 3:**
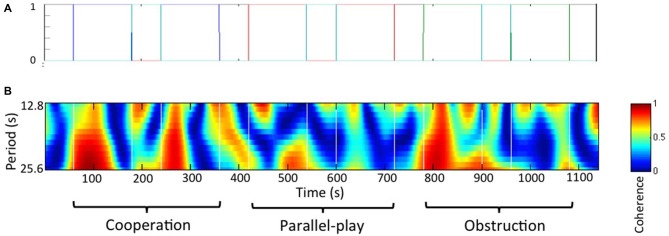
**Paired subjects wavelet coherences analysis. (A)** Regressors for general linear model (GLM) analysis. Each color line indicates an experimental condition and the black line indicates rest. **(B)** Wavelet coherence map for a representative dyad.

We used a general linear model (GLM) approach to study the effect of the task. The canonical GLM was introduced by Friston et al. (1994) and has been used extensively in analysis of functional MRI, as well as fNIRS in recent years. To apply GLM, the regressors are typically convolved with a hemodynamic response function (HRF) to better mimic the brain signal. However, in this hyperscanning project, we did not study the hemodynamic response evoked by a task for a single subject. Instead, we wanted to study the coherence of two hemodynamic responses, and evaluate whether an experimental manipulation evoked meaningful activation coherence. Thus, the regressors were not convolved with any HRF. Based on the task design, we created four regressors that were based on the onset time and duration of the four conditions separately (Figure 3A). The resulting beta values at each coherence channel were used in the following group analysis to determine the task effect at a group level.

### Node-Wise Random-Effect Group Analysis

For the group analysis, we developed a node-wise approach to identify common brain regions in each of the dyads, and then combined beta values from each common region into a single statistical test. Specifically, to identify common brain regions in dyads, we first generated uniformly distributed nodes on a spherical cap [Stefan Stoll, matlabCentral, stoll@phys.chem.ethz.ch], converted these nodes to MNI space, and projected them onto a cortical surface (Liu et al., 2015a). The generated nodes in MNI space were between 5–10 mm apart. We then projected all coherence channel positions from all dyads onto the same cortical surface. For each node, we counted how many dyads’ coherence channels were within its 15 mm diameter range. Nodes were then color-coded based on the number of dyads, and were mapped onto a MRI rendered brain template in MNI space.

The statistical analysis was only performed on those nodes that had eight or nine dyads’ coherence channels within their 15 mm diameter range. Those nodes were referred to as “effective nodes”. Thus, for each effective node, there was a set of associated coherence channels. When coherence channels within a set belonged to the same dyad, we averaged the beta values to generate a single (mean) beta value for the dyad at that effective node. We then performed a one-sample *t*-test of all corresponding dyads’ beta values for each effective node. A relatively conservative alpha value (*P* < 0.01) was used for each condition vs. rest, corrected with the Bonferroni method for multiple comparisons, to determine the inter-subject coherence on the cortical surface at the group level.

## Results

### Task Performance

**Figure 4:**
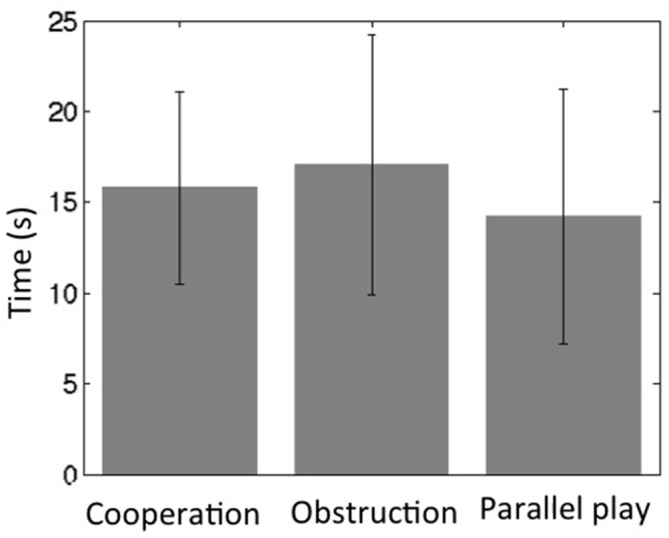
**Social interaction frequency during three task conditions (bars represent standard deviation)**.

All subjects were able to follow the instructions and maintained oral communication while playing the game. The towers remained standing during the cooperative and obstructive interaction conditions for all dyads, but fell for two subjects (two separate dyads) within the last 15 s of the parallel play condition. The average height of the tower at the end of the cooperation condition was 13.3 ± 0.44 cm, while the average height at the end of the parallel play condition was 13.7 ± 0.86 cm. The time intervals between two continuous movements of the wooden block across all dyads for the cooperation condition was 16 ± 5.3 s, for the obstruction condition 17 ± 7.1 s, and for the parallel play condition 14 ± 7.0 s (Figure 4). Two-sample *T*-tests revealed that there were no significant differences between the cooperation condition and the other two conditions for time interval (coop vs. obstr, *P* = 0.26; coop vs. para, *P* = 0.12). However, there was a significant time interval difference between the obstructive and parallel play conditions (*P* = 0.01).

### Functional Imaging

#### Paired Subject Wavelet Coherences

The frequency band for the wavelet coherences analysis was determined based on social interaction frequency of the dyads during the task, or the inverse of the time interval between two continuous movements of the wooden block. We chose the frequency between 0.08 Hz to 0.04 Hz (period between 12.8 s and 25.6 s) for this study based on the fact that our primary focus was on inter-brain synchrony during the cooperation and obstructive conditions. Thus, this frequency band was chosen as it best corresponded to activities during these conditions. A wavelet coherence map for a representative dyad is shown in Figure 3B.

#### Distribution of fNIRS measurement

**Figure 5:**
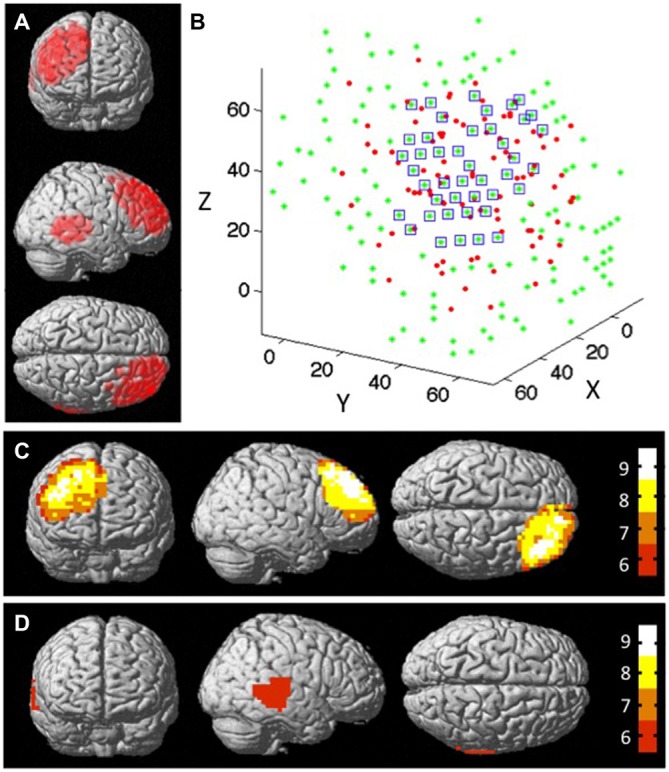
**The structural node-based spatial registration approach for inter-dyad analyses. (A)** The measured channel locations of all dyads on a MRI rendered brain template. **(B)** The measured channel locations of all dyads at rPFC region in 3D space. The red dots represent the effective locations of all dyads; the green dots represent the nodes that cover the measured cortical region; and the blue squares mark the nodes that have data from ≥8 subjects within 15 mm diameter distance from that node. Overlap map shows the distribution of the number of dyads that were measured by functional near-infrared spectroscopy (fNIRS): **(C)** at rPFC region, and **(D)** at rSTS region. Colorbar indicates the number of subjects that fall within 15 mm diameter distance from the node.

Figure 5A shows the distribution of all measured cortical locations across dyads using an MRI rendered brain template (xjView). This distribution covered the right prefrontal and right posterior temporal regions, which included the posterior portion of the rSTS. Figures 5C,D show the distribution of the number of dyads at the right prefrontal and right posterior temporal regions. The results indicate that the overlapping regions covered by at least eight dyads were mainly in the center of the overall measured regions. Outer edge regions were covered by less than eight dyads. The results also indicate that there were no regions in the rSTS that were covered by eight dyads’ measurements (Figure 5D). The green stars in Figure 5B show the distribution of all of the nodes within the rPFC. Among them, 43 were effective nodes, marked by blue squares in the figure.

#### Inter-Subject Cerebral Coherence During Cooperative Game Play

**Figure 6:**
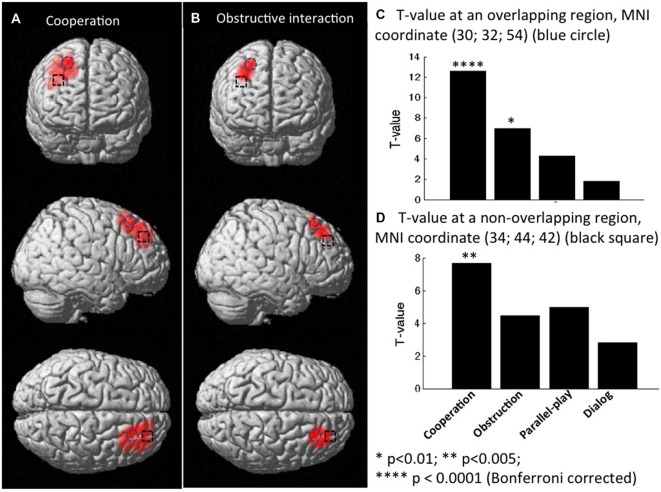
**Group *t*-maps for the contrast corresponding to coherence (A) in the cooperation condition vs. rest, and (B) in obstructive interaction condition vs. rest. (C)** *T*-values at a node that was significantly synchronized in both cooperation and obstruction conditions (vs. rest). **(D)** *T*-values at a node that was significantly synchronized only in the cooperation condition (vs. rest).

Group analysis was performed only on the effective nodes covered by at least eight dyads’ measurements. The rSTS region had no such effective nodes. This limited the group analysis to 43 effective nodes within the rPFC region and none in the STS region. We tested whether there was inter-subject cerebral coherence during each task condition vs. rest. We found strong coherence during both cooperative and obstructive conditions (*P* < 0.01, Bonferroni corrected), but not during the parallel play condition and the dialog section. There were 11 effective nodes associated with the cooperative condition and two effective nodes associated with the obstructive condition (Table 1, Figures 6A,B). The effective nodes associated with cooperative condition were located in the posterior region of middle and superior frontal gyrus, in particular Brodmann’s Areas 8 and 9 (BA8 and BA9). The effective nodes associated with the obstructive condition overlapped with two effective nodes associated with the cooperative condition, and were located in BA8 only. Figure 6C shows the *T*-values at an effective node in BA8 that was significantly synchronized for both cooperative and obstructive interaction conditions. Note that the *T*-value for the cooperative condition is larger than that of the obstructive interaction condition. Figure 6D shows the *T*-values at an effective node in BA9 that was significantly synchronized for the cooperative condition but not for the obstructive interaction condition.

**Table 1:** **Foci of significant effective nodes associated with cooperative and obstructive conditions**.

Region	Brodmann area	MNI coordinates			*Z* score	*P* value
		*x*	*y*	*z*		(Bonferroni corrected)
*Cooperative condition*
Superior frontal gyrus	8	22	48	46	2.65	0.0041
Superior frontal gyrus	8	26	40	50	2.98	0.0014
Superior frontal gyrus	8	30	28	58	2.94	0.0016
Superior frontal gyrus	8	30	32	54	3.83	0.0001
Superior frontal gyrus	8	34	24	58	2.86	0.0021
Superior/Middle	8	34	40	46	3.47	0.0003
frontal gyrus
Middle frontal gyrus	9	34	44	42	2.80	0.0025
Superior/Middle	8	38	32	50	3.01	0.0013
frontal gyrus
Middle frontal gyrus	8	38	36	46	2.61	0.0045
Middle frontal gyrus	8	42	28	50	2.49	0.0063
Middle frontal gyrus	9	42	44	34	2.38	0.0087
*Obstructive condition*
Superior frontal gyrus	8	30	32	54	2.58	0.0050
Superior/Middle	8	34	40	46	2.42	0.0078
frontal gyrus

**Figure 7:**
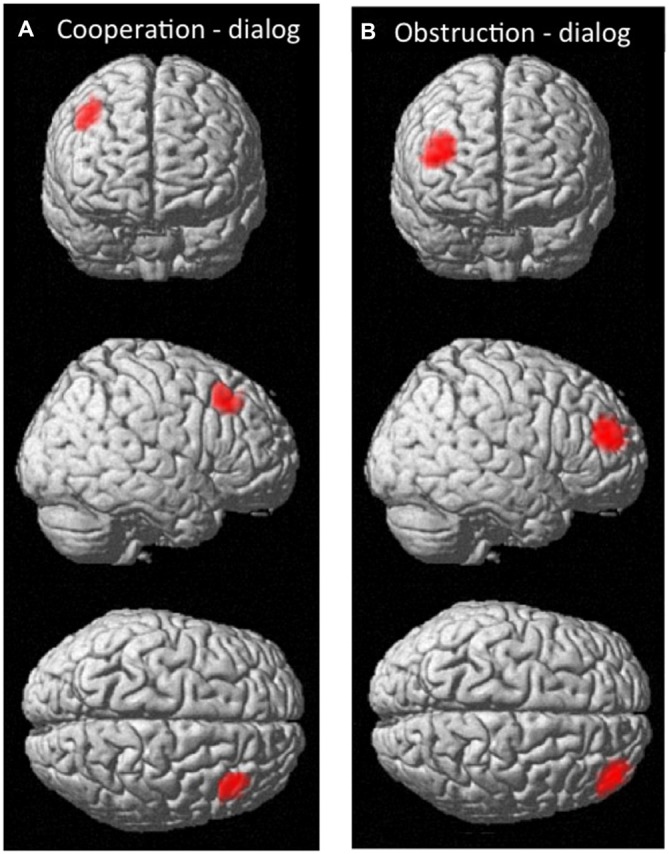
**The effective nodes for the contrast corresponding to coherence (A) in the cooperation vs. dialog condition; (B) in the obstructive interaction vs. dialog condition**.

We also tested whether there was significant inter-subject cerebral coherence difference between any two conditions. There were no nodes that showed statistically significant between-condition coherence differences at the group level. However, based on uncorrected P values, two regions of potential interest to human social cognition were observed (Figure 7). One region (MNI coordinates: 46, 28, 46) located at the middle frontal gyrus (BA8) showed a trend for differentiating coherence during the cooperation vs. dialog contrast (cooperation > dialog, *t* = 3.02, *P* = 0.017, uncorrected; Figure 7A). Similarly, another region (MNI coordinates: 42, 52, 22 and 46, 48, 22) located at middle frontal gyrus (BA10) showed a trend for differentiating coherence during the obstructive vs. dialog contrast (obstruction > dialog, [46 48 22]: *t* = 3.40, *P* = 0.009, uncorrected; [42 52 22]: *t* = 3.43, *P* = 0.011, uncorrected; Figure 7B). Both BA 8 and 10 are thought to be involved in social cognition, especially as occurring between two people (McCabe et al., 2001; Grimes, 2003; Chorvat and McCabe, 2004). Further studies with a larger sample size and appropriately corrected significance threshold are needed to further explore these findings.

## Discussion

It has been realized that “an important step forward in social neuroscience is the development of paradigms allowing for the study of such true, real-time social interactions” (Singer, 2012). Our study was an effort to address this limitation by establishing a novel experimental design that models naturalistic interdependent social behavior and communicative interactions between people.

All participants were able to follow the instructions based on the observation of the operators. Participants refrained from interaction during the parallel play condition and engaged in appropriate interaction during other conditions. The average time interval between two continuous movements of the wooden block was longer for the cooperation and obstruction conditions than for parallel play condition. This indicates that the participants understood the instructions and implemented some form of interaction during the appropriate conditions.

Strong inter-brain synchrony was observed at two effective nodes in the middle and superior frontal gyrus (BA8) during the cooperative and obstructive interaction conditions, but not during the parallel play condition and the dialog section. This finding suggests that BA8 cortical regions were involved in goal-oriented social interaction in our experiment. Studies have been shown that BA8 is active when individuals plan complex movements, make decisions that are uncertain, and use prospective memory to remember to do something in the future (Okuda et al., 1998; Fincham et al., 2002; Volz et al., 2005). In our turn-based study, individuals in the dyad were continuously making decisions based on their partner’s suggestions and responses during the cooperation and obstructive conditions, but not during the parallel play condition. That is to say, our results suggest that the observed coherence in this area might be a feature of social decision-making when two people interact. A recent study by Tang et al. (2016) investigated the INS in the right dorsolateral prefrontal cortex (rDLPFC) during an economic exchange task that involved oral communication within dyads. They used the channel-wise data analysis method, and the results indicated increased (but not significant) synchronization in rDLPFC channels (Figure 3C in Tang et al. 2016). The difference between Tang’s results and the results reported here might indicate that BA8 was involved in complex interactive movements but not pure dyadic communication in social cognition.

Inter-brain synchrony was observed at nine additional effective nodes in dorsomedial prefrontal cortex (dmPFC; BA9) only during the cooperative interaction condition. The dmPFC is thought to be part of the brain network that is activated by considering the intentions of another individual in social processing (Behrens et al., 2008, 2009). This region has been demonstrated to be active during “theory of mind” (ToM) games played with other individuals, but not played with computers (Greene and Haidt, 2002; Amodio and Frith, 2006; Saxe, 2006). During the cooperative condition in our version of the Jenga game, two players were instructed to communicate and agree on each movement before they physically moved the wooden block, which meant they had to fully understand their partner in order to reach the goal of building the wooden tower as tall as possible. This interaction was associated with increased inter-brain synchronization. However, in the obstructive condition, participants may have been less likely to fully regard or consider their partner’s recommendations when making their final decision on block movement. When combined with previous findings, our results suggest the involvement of dmPFC (BA9) in cooperative social behavior.

It is noteworthy that the coherence region associated with cooperation (covered by 11 effective nodes total) was much larger than that associated with the obstructive interaction (covered by two effective nodes). This indicates that cooperative interaction recruited more prefrontal regions than the obstructive interaction in this turn-based Jenga game. This finding might also be associated with task difficulty in that the cooperation condition might have been harder than the obstruction condition.

In this study we also developed a method to identify synchronized channels within a dyad and a structural node-based spatial registration approach for inter-dyad analyses. Compared with existing methods, this new approach does not require a structural MRI for each participant, does not need interpolation, and allows for preservation of the spatial resolution of the fNIRS imaging system. More importantly, the approach described here can help to offset the inevitable variability in fNIRS probe placement on an individual subject’s head, making it possible to combine data from multiple dyads to a common stereotaxic space for group analyses.

There were several limitations to our study. First, our sample size was small, and we were able to cover only a portion of the brain given the number of channels available with our fNIRS instrument. Therefore, important parts of the rSTS might not have been captured for some dyads, and we were unable to perform statistical analysis for that region. Additionally, during the group analysis, we performed a Bonferroni correction based on *n* = 43 (total number of effective nodes). However, based on our definition of an effective node there was likely inter-node (spatial) dependence and thus, the method of correction based on the number of nodes may have been too conservative by overestimating the number of independent spatial units. Third, we only videotaped five dyads, which limited our ability to analyze behavioral data and assess associations between these data and fNIRS activation. Fourth, the choice of values for the upper and lower limits of the frequency band for the WTC analysis was relatively arbitrary. Future research could focus on a more quantitative method to determining these values.

## Conclusion

In summary, the present study reveals inter-brain neural synchronization in the prefrontal cortex, in particular BA8 and BA9, during a naturally occurring Jenga game with face-to-face communication. Inter-brain synchrony was observed in BA8 during both cooperative and obstructive interaction conditions but not during the parallel play condition and the dialog section. These results indicate that BA8 plays a role in goal-oriented social interactions such as complex interactive movements and social decision-making. Inter-brain synchrony was also observed in BA9 during the cooperative interaction condition but not during the obstructive interaction and the parallel play conditions. These additional findings suggest that BA9 may be particularly engaged when ToM is required for cooperative social interaction. In addition, we also developed a method to identify synchronized channels for each dyad and a structural node-based spatial registration approach for inter-dyad analysis. These novel methods have the potential to significantly extend fNIRS hyperscanning applications to social cognitive research.

## Author Contributions

NL, CM, EEW and AHP: study design, collect data, data analysis, manuscript preparation. JEC: data analysis, manuscript preparation. ALR: study design, manuscript preparation.

## Conflict of Interest Statement

The authors declare that the research was conducted in the absence of any commercial or financial relationships that could be construed as a potential conflict of interest.

## Footnotes

^1^http://www.alivelearn.net/xjview
